# Spectral analysis of physiological brain pulsations affecting the BOLD signal

**DOI:** 10.1002/hbm.25547

**Published:** 2021-05-26

**Authors:** Lauri Raitamaa, Niko Huotari, Vesa Korhonen, Heta Helakari, Anssi Koivula, Janne Kananen, Vesa Kiviniemi

**Affiliations:** ^1^ Oulu Functional Neuro Imaging Group, Research Unit of Medical Imaging Physics and Technology (MIPT) University of Oulu Oulu; ^2^ Department of Diagnostic Radiology, Medical Research Center (MRC) Oulu University Hospital Oulu

**Keywords:** amplitude of low‐frequency fluctuation, cardiorespiratory modulation, fast fMRI, global signal, physiological brain pulsations, resting state

## Abstract

Physiological pulsations have been shown to affect the global blood oxygen level dependent (BOLD) signal in human brain. While these pulsations have previously been regarded as noise, recent studies show their potential as biomarkers of brain pathology. We used the extended 5 Hz spectral range of magnetic resonance encephalography (MREG) data to investigate spatial and frequency distributions of physiological BOLD signal sources. Amplitude spectra of the global image signals revealed cardiorespiratory envelope modulation (CREM) peaks, in addition to the previously known very low frequency (VLF) and cardiorespiratory pulsations. We then proceeded to extend the amplitude of low frequency fluctuations (ALFF) method to each of these pulsations. The respiratory pulsations were spatially dominating over most brain structures. The VLF pulsations overcame the respiratory pulsations in frontal and parietal gray matter, whereas cardiac and CREM pulsations had this effect in central cerebrospinal fluid (CSF) spaces and major blood vessels. A quasi‐periodic pattern (QPP) analysis showed that the CREM pulsations propagated as waves, with a spatiotemporal pattern differing from that of respiratory pulsations, indicating them to be distinct intracranial physiological phenomenon. In conclusion, the respiration has a dominant effect on the global BOLD signal and directly modulates cardiovascular brain pulsations.

## Graphical abstract

Extensive 5 Hz spectral resolution of fast fMRI revealed a new form of physiological brain contrast illustrating respiratory‐induced modulation of cardiovascular brain pulsations. Very low frequency (VLF), respiratory and cardiovascular pulses, their harmonics and the detected modulations were found to form the global BOLD signal. Propagating respiratory pulsations should be removed cautiously with voxel‐wise Fourier filtering as they compete globally with every other BOLD signal source.

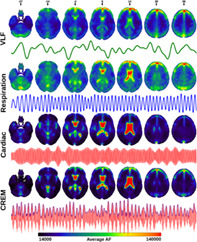

## 1 INTRODUCTION

Roy and Sherrington noted in their pioneering 1890 study on animal cerebral hemodynamics that, in addition to hemodynamic coupling to electrical stimuli, “the brain expands with each rise of the blood pressure and contracts with each successive fall” during the frequently detected spontaneous Mayer blood‐pressure waves (Roy & Sherrington, 1890). A decade later, Hans Berger showed that there were three sources of intracranial brain pressure pulsations, namely the *“* pulsatory, respiratory and vasomotor waves” (Berger, 1901). To this day, an understanding of the physiological significance of these pulsations remains elusive. In contemporary studies, conventional fMRI uses blood oxygen level dependent (BOLD) signals to measure hemodynamic changes following neuronal brain activity that can be either cued or spontaneous in nature (Bandettini, Wong, Hinks, Tikofsky, & Hyde, 1992; Fox & Raichle, 2007; Ogawa, Lee, Kay, & Tank, 1990). The BOLD signal changes are also affected by the presence of broadly synchronous fluctuations, termed as the global signal, which is thought to contain undesired variance from non‐neural sources. Therefore, regression of a global signal from each voxel is a common method to remove these signals in a preprocessing step for fMRI data (Erdoğan, Tong, Hocke, Lindsey, & deB Frederick, 2016; Fox & Raichle, 2007; Giove, Gili, Iacovella, Macaluso, & Maraviglia, 2009; Macey, Macey, Kumar, & Harper, 2004). However, the exact origin of the global signal is unclear, although it is often attributed to physiological noise (Liu, Nalci, & Falahpour, 2017; Murphy & Fox, 2017; Power, Plitt, Laumann, & Martin, 2017). Indeed, it has been difficult to ascribe exact physiological source of these signals due to the problem of signal aliasing and further spatiotemporal mixing from interleaved data sampling the BOLD signal, which is usually sampled at a low frequency.

On the other hand, the recent discovery of the glymphatic brain clearance pathway driven by brain pulsation has increased the relevance of physiological pulsations, which are emerging as a signal of interest instead of a mere nuisance noise source. In the glymphatic system, the physiological pulsations have been shown to drive water and brain metabolite convection along perivascular spaces and within the brain interstitium in both mice and men (Kiviniemi et al., 2016; Meng et al., 2019; Mestre et al., 2018; Wang et al., 2017). Importantly, a failure of glymphatic convection has been shown to precede the onset of neurodegeneration (Iliff et al., 2012) and to affect the clinical trajectory of several diseases such as epilepsy, trauma, and stroke (Lin et al., 2020; Liu et al., 2020; Rasmussen, Mestre, & Nedergaard, 2018; Sullan, Asken, Jaffee, DeKosky, & Bauer, 2018).

In line with the emergent concept of glymphatics, physiological variance of the BOLD signal has increasingly been related to pathology (Garrett, Kovacevic, McIntosh, & Grady, 2010; Helakari et al., 2019; Hussein et al., 2020; Jahanian, Peltier, Noll, & Garcia, 2015; Kananen et al., 2018, 2020; Makedonov, Chen, Masellis, & MacIntosh, 2016). The cardiorespiratory pulsations have been shown to drive blood and also cerebrospinal fluid (CSF) flow inside the brain, and measures of these pulsations thus contain valuable information on flow dynamics (Dreha‐Kulaczewski et al., 2015; Kiviniemi et al., 2016). For example, there is a significant alteration in the variance of cardiovascular pulsation in the brain of Alzheimer' disease patients (Rajna et al., 2019, 2021; Tuovinen et al., 2020).

The increasing interest in physiological brain pulsations from the perspective of noise correction also has implications for clinical diagnostics. Both perspectives call for greater in‐depth knowledge on how to characterize accurately brain pulsations. In this study, we investigated physiological signal sources affecting whole brain BOLD signals using a high temporal resolution 3D brain scanning with magnetic resonance encephalography (MREG). The technique of MREG enables critical separation of the higher frequency physiological pulsations from the VLF signal without any need for aliasing (Huotari et al., 2019; Kiviniemi et al., 2016; Tuovinen et al., 2020). We used the extended 5 Hz spectral resolution of our method to investigate and localize physiological sources of BOLD signal variance. In addition to confirming previously known physiological pulsations, we detected by this means a novel cardiorespiratory envelope modulation (CREM). We proceeded to map the amplitudes of the various physiological pulsations over the whole brain using a modified ALFF method (Yu‐Feng et al., 2007). Finally, we applied QPP analysis to compare propagation patterns of the modulatory CREM brain wave with respect to its effects on respiratory brain waves and conclude with a discussion of their role in the generation of the whole brain BOLD signal.

## 2 MATERIALS AND METHODS

### 2.1 Participants

Fifty‐three healthy subjects (age: 40.5 ± 17.0 years, 32 females) entered the MRI scanner and were instructed to lie still, with eyes kept open and gaze fixated on a cross on the screen while thinking of anything in particular (eyes open, resting state). Ear plugs were provided to reduce scanner noise. Cushions were placed beside the ears to restrict head movement and to further reduce scanner noise. During image preprocessing, three subjects were excluded because of partial data corruption and two because of excess head motion. Data from the remaining 48 subjects (age: 40.7 ± 17.2 years, 29 females) were used in this study. Written informed consent was obtained from each subject prior to scanning, in accordance with the Helsinki declaration. The study protocol was approved by the regional Ethical committee of Northern Ostrobothnia Hospital District in Oulu University Hospital.

### 2.2 Data acquisition and preprocessing

Subjects were scanned using a Siemens MAGNETOM 3 T SKYRA scanner with a 32‐channel head coil. Additional cardiorespiratory data were collected using an MRI‐compatible multimodal neuroimaging Hepta‐Scan concept (Korhonen et al., 2014). MREG is a 3D single shot stack of spirals (SOS) sequence that under‐samples k‐space to reach a sampling rate of 10 Hz, thus allowing critical imaging of physiological pulsations (Assländer et al., 2013). The SOS gathers k‐space in 60 ms bins with spiral in/out repeating in every other turn continuously in the positive z‐direction, thus minimizing the air‐sinus off‐resonance artifact (Assländer et al., 2013). The point spread function of the SOS‐sequence is 3 mm, with lesser off‐resonance effects compared to other k‐space undersampling strategies such as concentric shells and spokes (Assländer et al., 2013; Zahneisen et al., 2012). Scanning parameters were TR = 100 ms, TE = 36 ms, flip angle = 25°,3D matrix = 643, FOV = 192 mm with voxel size of 3 × 3 × 3 mm^3^, and for anatomical 3D MPRAGE the parameters were TR = 1900 ms, TE = 2.49 ms, TI = 900 ms, flip angle = 9, FOV = 240 mm, 0.9 mm cubic voxel. Scans lasted only 5 min. Cardiorespiratory frequencies were verified with an anesthesia monitor (GE Date‐Ohmeda Aestive 5) and from scanner physiological data recordings.

MREG data were reconstructed using L2‐Tikhonov regularization with lambda 0.1, where the latter regularization parameter was determined by the L‐curve method with a MATLAB recon‐tool from the sequence developers (Hugger et al., 2011). T1‐relaxation effects were minimized by deleting the 14 s from the beginning of each scan. The AFNI *3dDespike* function (with options—NEW and—localedit) was used to remove spikes from the remaining data. Then data were preprocessed with the standard FSL (Functional Magnetic Resonance Imaging of the Brain's software library) pipeline (Jenkinson, Beckmann, Behrens, Woolrich, & Smith, 2012), using high‐pass filtration with a cut‐off frequency of 0.008 Hz (125 s). Motion correction was performed using FSL MCFLIRT, and FSL BET was used for brain extraction. The anatomical 3D MPRAGE images were used to register MREG data into MNI152 standard space using FSL FLIRT (Grabner et al., 2006; Jenkinson et al., 2012).

As this study focuses on the sources of physiological BOLD signals, we wanted as much as possible to retain the physiological pulsations in the data. Therefore, CSF, white matter and global signals were not regressed out of the datasets. Spatial smoothing was also omitted since it averages the signal between neighboring voxels, thus degrading spatial resolution of the detection of original pulsation signals, and this factor of unsmoothed data is also taken into account in the recently developed LIPSIA‐tool that we used to draw more accurate statistical inferences (Lohmann et al., 2018; Wu et al., 2011).

### 2.3 Theoretical basics of modulation

Modulation is the act of translating information from a low‐frequency signal to a higher frequency. In the modulation process, amplitude, frequency and/or phase of a high‐frequency signal is changed in direct proportion to the instantaneous values of the low‐frequency signal (Writer, 1999). In amplitude modulation, the low‐frequency signal modifies and scales the amplitude of the high‐frequency signal and determines the envelope of the waveform.

**FIGURE 1:**
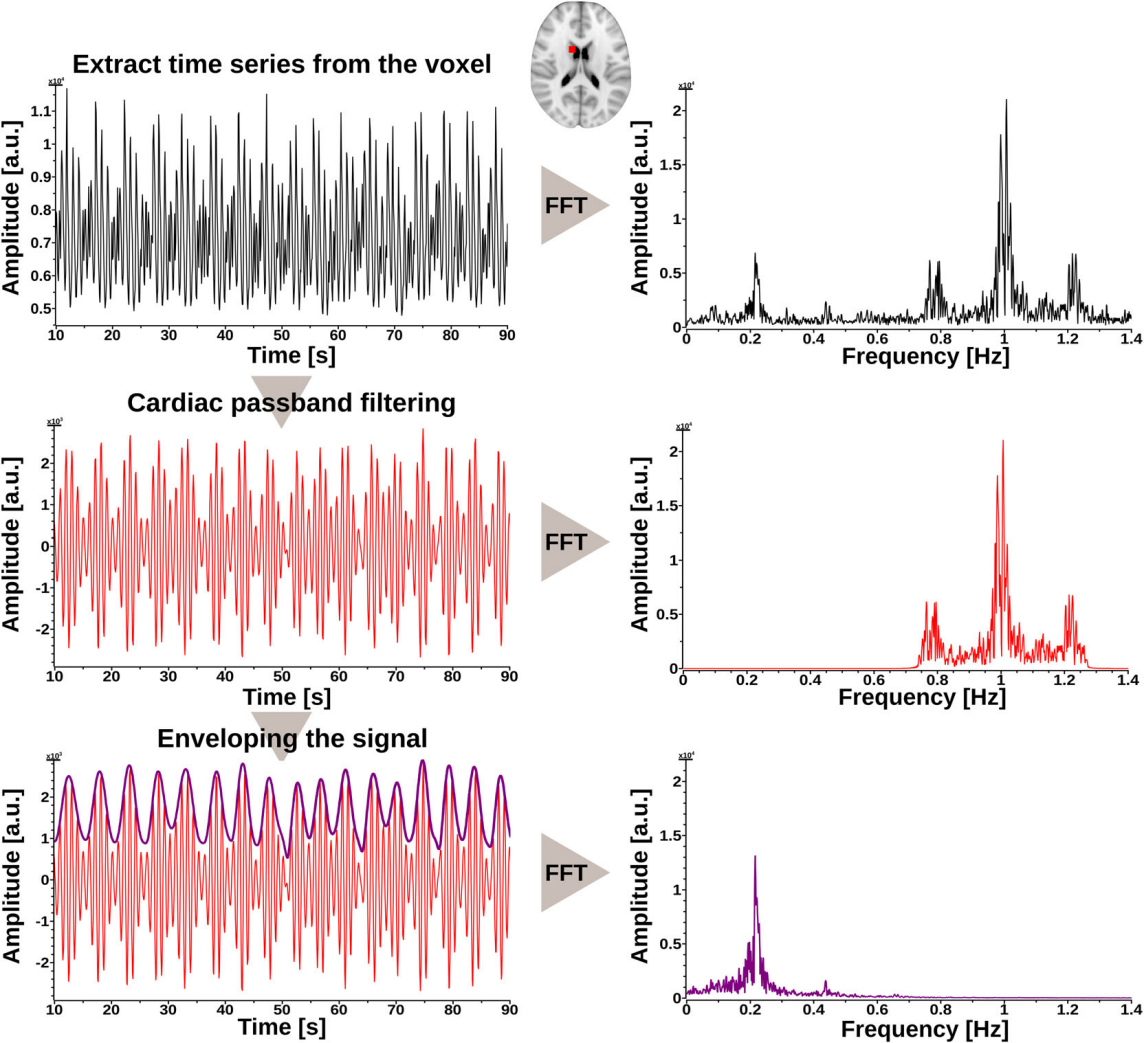
Time series analysis of cardiorespiratory envelope modulation (CREM). Analysis steps from a representative subject from a 3 mm diameter spherical region of interest ROI (AP, etc., 12, 6, 18 mm in MNI space) (Left) Time series extracted from the ROI. Extracting mean time series from the mask (black signal)— >band‐pass filtering in cardiac band (red signal)— > taking upper envelope from cardiac bandpass filtered signal to acquire CREM (purple signal) (Right) Corresponding fast Fourier transformation (FFT) spectrums for every time series during analysis

**FIGURE 2:**
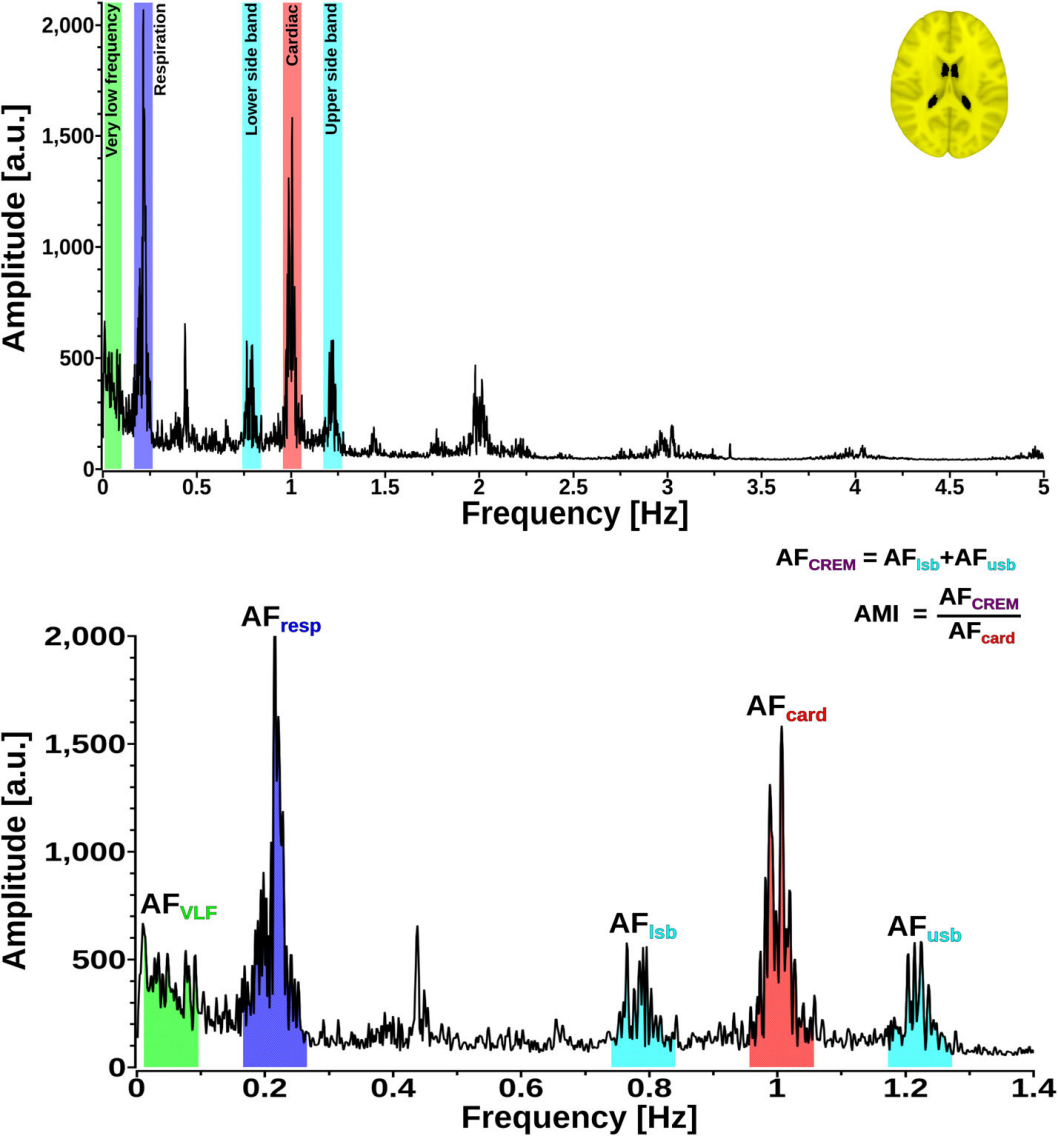
Illustration of the analysis methods: (Upper) A representative whole‐brain full‐band 0–5 Hz fast Fourier transformation (FFT) spectrum. (Bottom) A focused 1.4 Hz FFT spectrum window from the upper spectrum showing bands taken for calculating amplitude fluctuations (AF) analysis

The method of detecting CREM is based on the heterodyne principle; multiplied sinusoidal waveforms can be written as the sum and the difference of the applied frequencies. In the case of amplitude modulation, we can detect sidebands on either side of the fundamental band (Figure 1). The distance between the lower/upper sidebands peak and fundamental band peak is equal to the modulating signal respiratory frequency. In our case, we detect two sidebands around the fundamental cardiac frequency at a distance equal to the respiratory frequency (Figures 1 and 2). By defining an envelope of the cardiac signal, we can obtain a time signal for CREM, which has a frequency component equal to the respiration frequency (Figure 1, purple envelope).

### 2.4 Analysis of amplitude fluctuation of physiological signals

The MREG pulsation data have been shown to match accurately the real physiological pulsations measured simultaneously with monitoring devices (Kiviniemi et al., 2016; Raitamaa et al., 2018; Tuovinen et al., 2020). For every subject, the time courses of each voxel from whole brain MREG data were transformed using AFNI *3dPeriodogram* to the frequency domain via a fast Fourier transformation, which yielded the voxel‐wise power spectrum. A global power spectrum was then summed from all brain voxels to investigate detectable physiological pulsation bands affecting the global BOLD signal. From the MREG data spectrum, we obtained frequency peaks of respiratory (mean: 0.25 ± 0.06 Hz) and cardiac pulsations (mean: 1.10 ± 0.14 Hz). These MREG signal pulsations frequencies were verified from the SpO2 and respiratory data from the simultaneously used anesthesia monitor.

We next used a modified ALFF method to study amplitude fluctuation (AF) of very low frequency (AF_VLF_), respiratory (AF_resp_), and cardiac (AF_card_) pulsations (Yu‐Feng et al., 2007). The frequency band for AF_VLF_ was 0.01–0.1 Hz, corresponding to the classical ALFF, while the bands for AF_resp_ and AF_card_ were 0.1 Hz wide, centered around the previously defined individual peaks (i.e., peak ±0.05 Hz indicated in colors; Figure 2). The square root of the power spectral density was calculated, and amplitudes calculated over the frequency bands of interest were summed to obtain a corresponding AF map. The AF value in a given voxel represents the total voxel‐wise amplitude of a chosen frequency band, which reflects the local features of brain oscillatory activities (Yu‐Feng et al., 2007; Zou et al., 2008).

**FIGURE 3:**
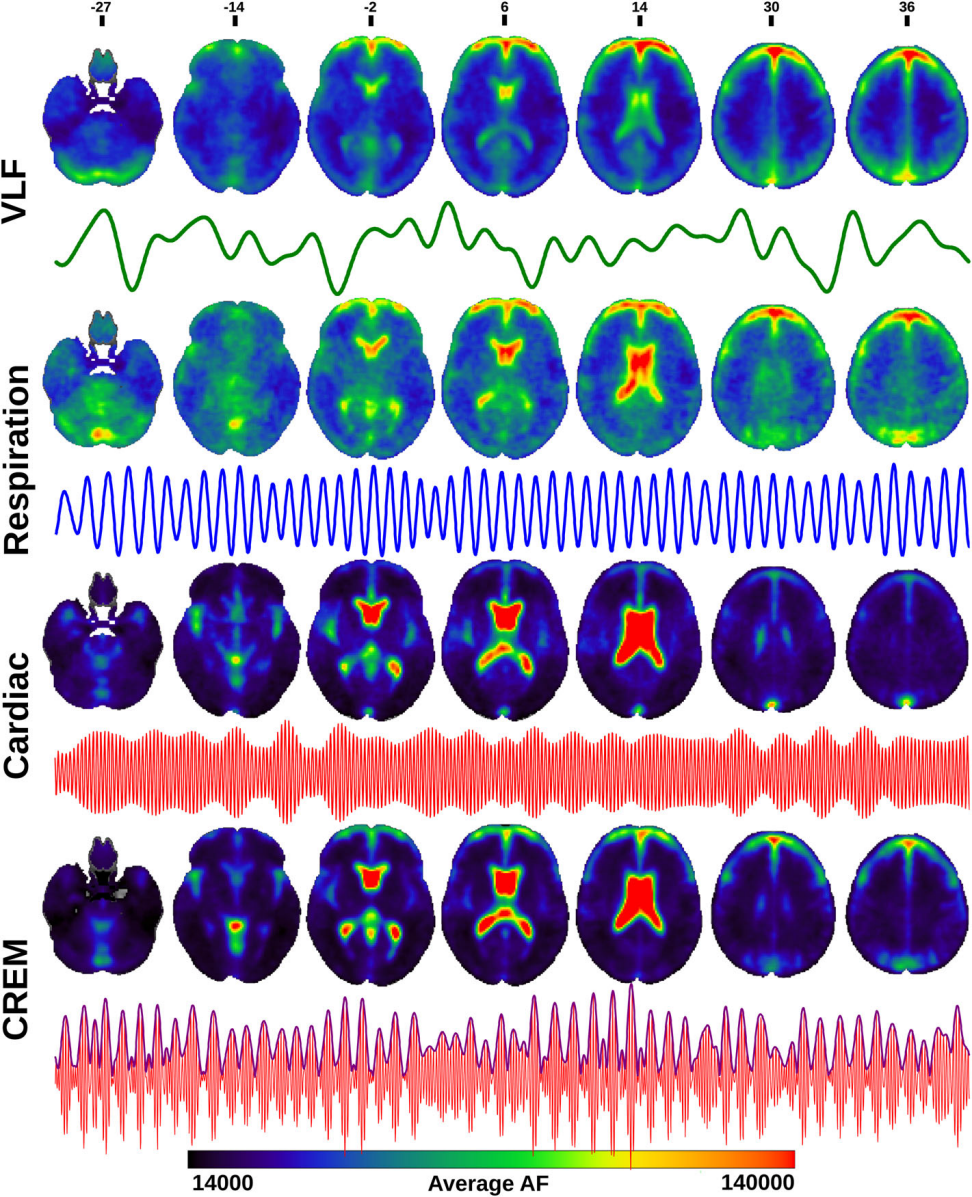
Amplitude fluctuation (AF) images: Group averages of AF maps in the four different bands: very low frequency (VLF), respiration, cardiac and cardiorespiratory envelope modulation (CREM), and 5‐min representative time‐series signals from the anterior right cerebral white matter (VLF), right lateral ventricle (Respiration and cardiac), and posterior left cerebral white matter (CREM) for each band of a representative individual subject: VLF (green), respiration (blue), cardiac (red) and CREM (purple). Color bar indicates the AF range of all group average maps

The amplitude fluctuations of the lower and upper sidebands (AF_lsb_ and AF_usb_) were quantified according to heterodyne principle based on the respiratory and cardiac peaks, using the same band widths of 0.1 Hz. The amplitude of cardiorespiratory modulation (AFCREM) was calculated as the sum of AF_lsb_ and AF_usb_, c.f. Figure 2. Group average maps for every AF band were calculated (Figure 3).

To quantitate the relative strength of modulation and to remove any possible scaling effects that could affect AF_CREM_ and/or AF_card_, we also calculated a commonly used signal analysis metric called amplitude modulation index (AMI), which defines a ratio of the modulated CREM signal with respect to the unmodulated cardiovascular pulsation signal (AMI = AF_CREM_/AF_card_; Figure 2) (Writer, 1999). AMI is normalized and unitless as it gives a percentage of the modulation and tells how much the modulated variable of the signal varies around its unmodulated level.

**FIGURE 4:**
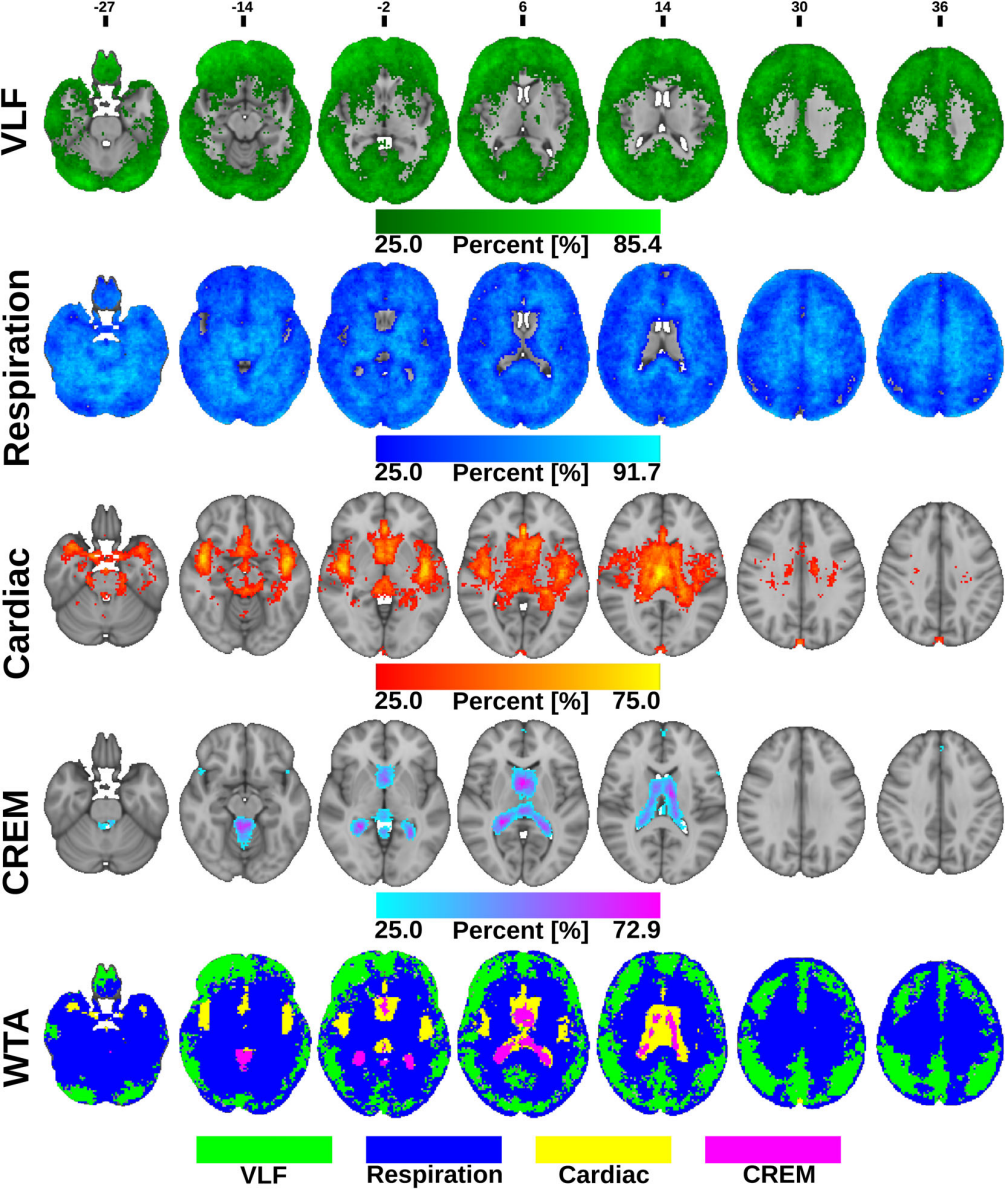
Proportional whole brain maximum amplitude maps at each band: From top to bottom, the very low frequency (VLF), respiration, cardiac, and cardiorespiratory envelope modulation (CREM). Colors represent the number of subjects per voxel where the corresponding pulsation frequency dominates over other frequencies, range: from 12 to the maximum number of subjects per frequency. Winner takes all (WTA) map at the bottom indicates which physiological pulsation dominates over others in each voxel of the brain

To investigate the relative spatial dominance of each pulsation source that dominates over the other frequencies in the brain, we first created subject‐specific maps where each voxel was assigned to one of the four pulsation bands defined by which band had the highest AF value. After this procedure, we created proportional brain maps for every frequency band over all imaged subjects by calculating the number of subjects per voxel where the amplitude of the corresponding frequency exceeded that in any other frequency and dividing the number of total number of subjects (Figure 4). Voxels, where at least 25% of subjects have the same dominant frequency, were mapped. Finally, we made a winner takes all (WTA) map, wherein every voxel was assigned to one of the four band based on which band had the most subjects; in the rare event of tie, that voxel was not assigned to any band (Figure 4).

### 2.5 Statistical analysis

**FIGURE 5:**
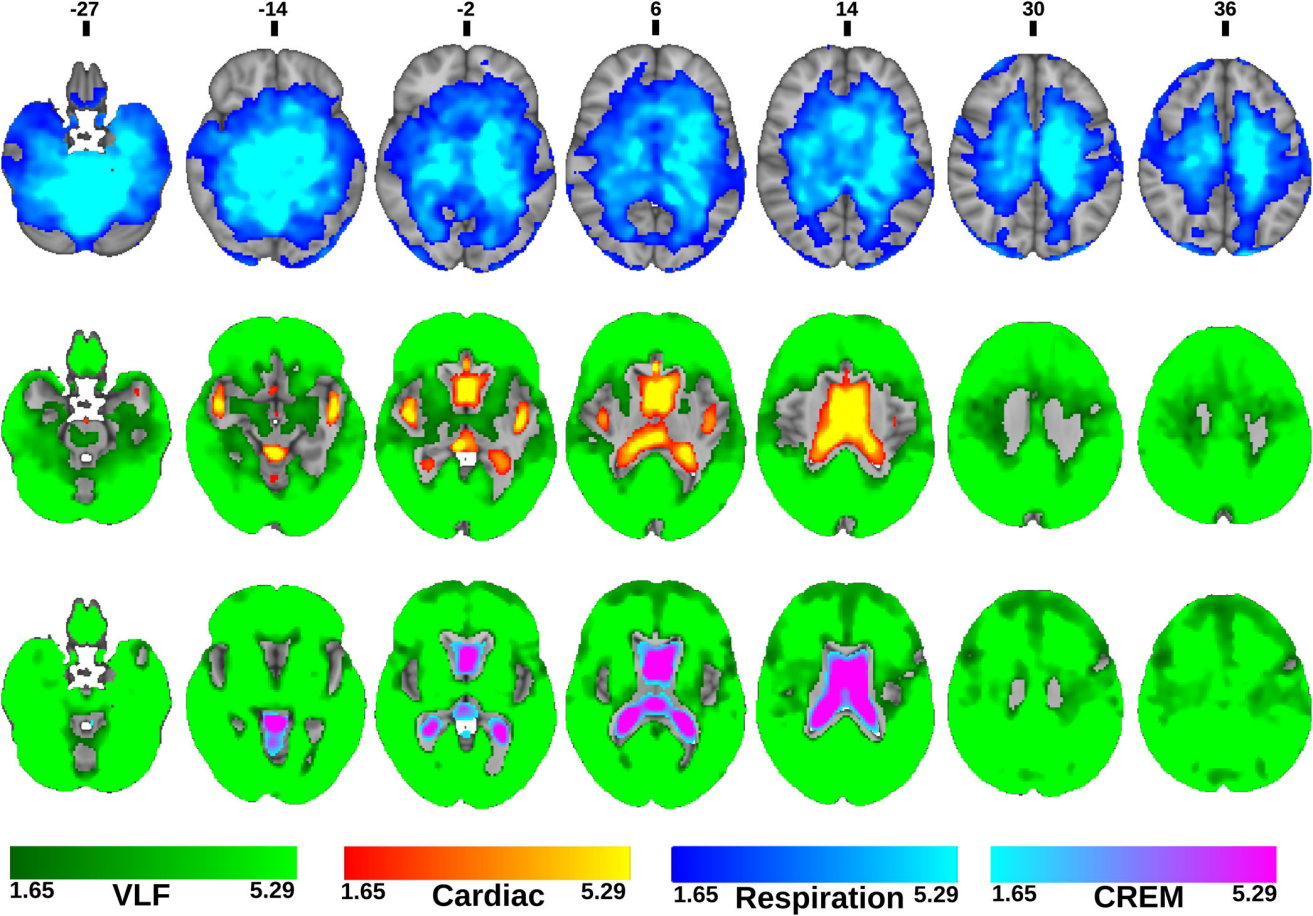
Statistical amplitude of very low frequency fluctuation (AF_VLF_) comparison maps. Statistical comparison of AF_VLF_ to amplitude of respiratory fluctuation (AF_resp_)(top), amplitude of cardiac fluctuation (AF_card_) (middle) and amplitude of cardiorespiratory envelope modulation (AF_CREM_) (bottom). Colors in the maps represent where AF_card_ (Red), AF_resp_ (blue) or AF_CREM_ (Purple), respectively, are higher compared to AF_VLF_. (green) indicates where AF_VLF_ dominates over the compared frequency. (FDR corrected, *p* < .05 with respective z‐score encoding). Note the missing difference in the amplitudes between respiration and VLF in the cortex

**FIGURE 6:**
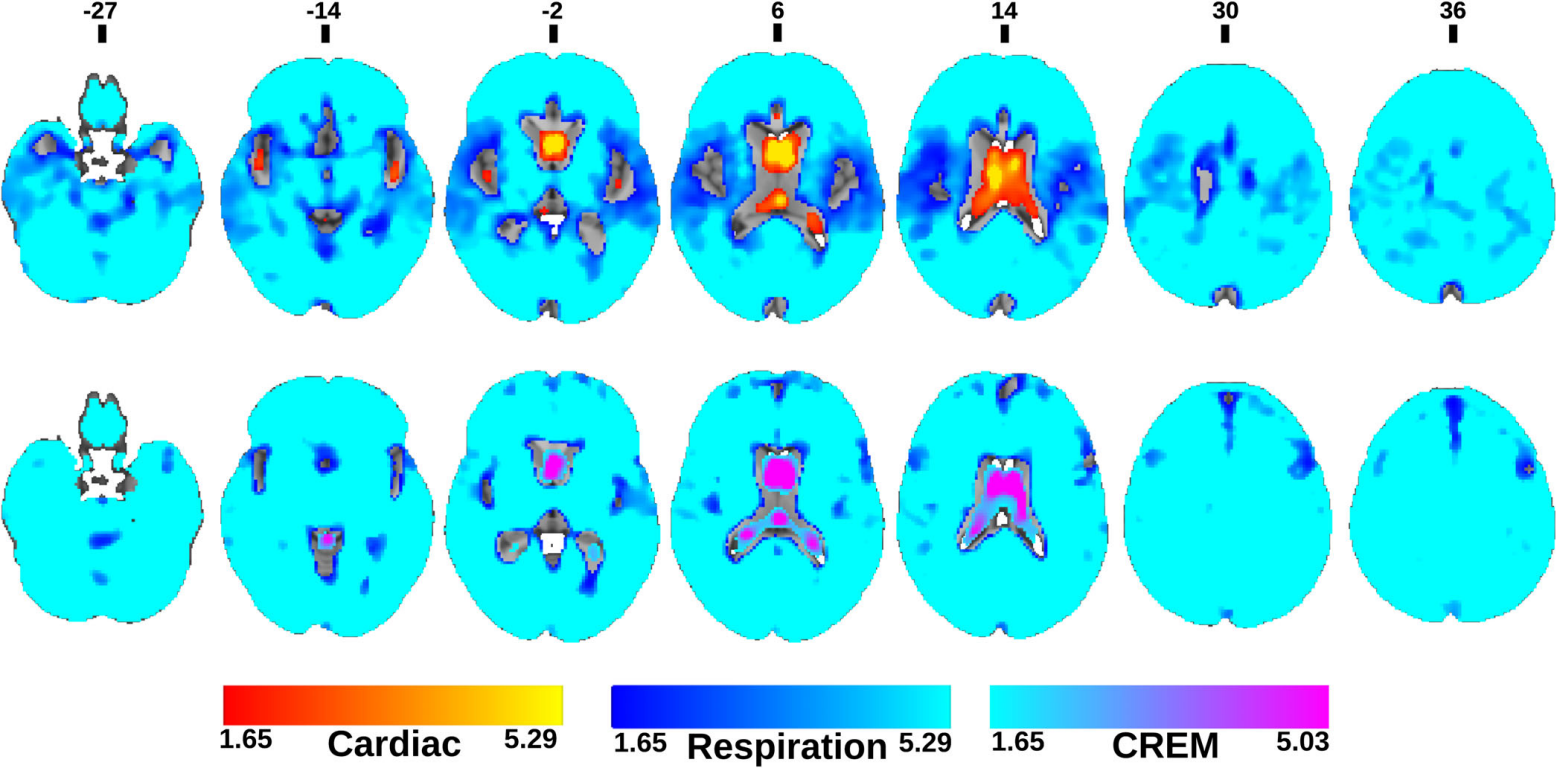
Statistical amplitude of respiratory fluctuation (AF_resp_) comparison maps. Statistical comparison of AF_resp_ to amplitude of cardiac fluctuation (AF_card_) (top) and amplitude of cardiorespiratory envelope modulation (AF_CREM_) (bottom). Colors in the maps represent areas where AF_card_ (Red) or AF_CREM_ (Purple), respectively are higher compared to amplitude of respiratory fluctuation (AF_resp_) (Blue), and vice versa. (FDR corrected, *p* < .05 with respective z‐score encoding). Comparison between amplitude of very low frequency fluctuation (AF_VLF_) and AF_resp_ was done in the previous Figure 5. The respiratory pulsation dominates widely in the brain

**FIGURE 7:**
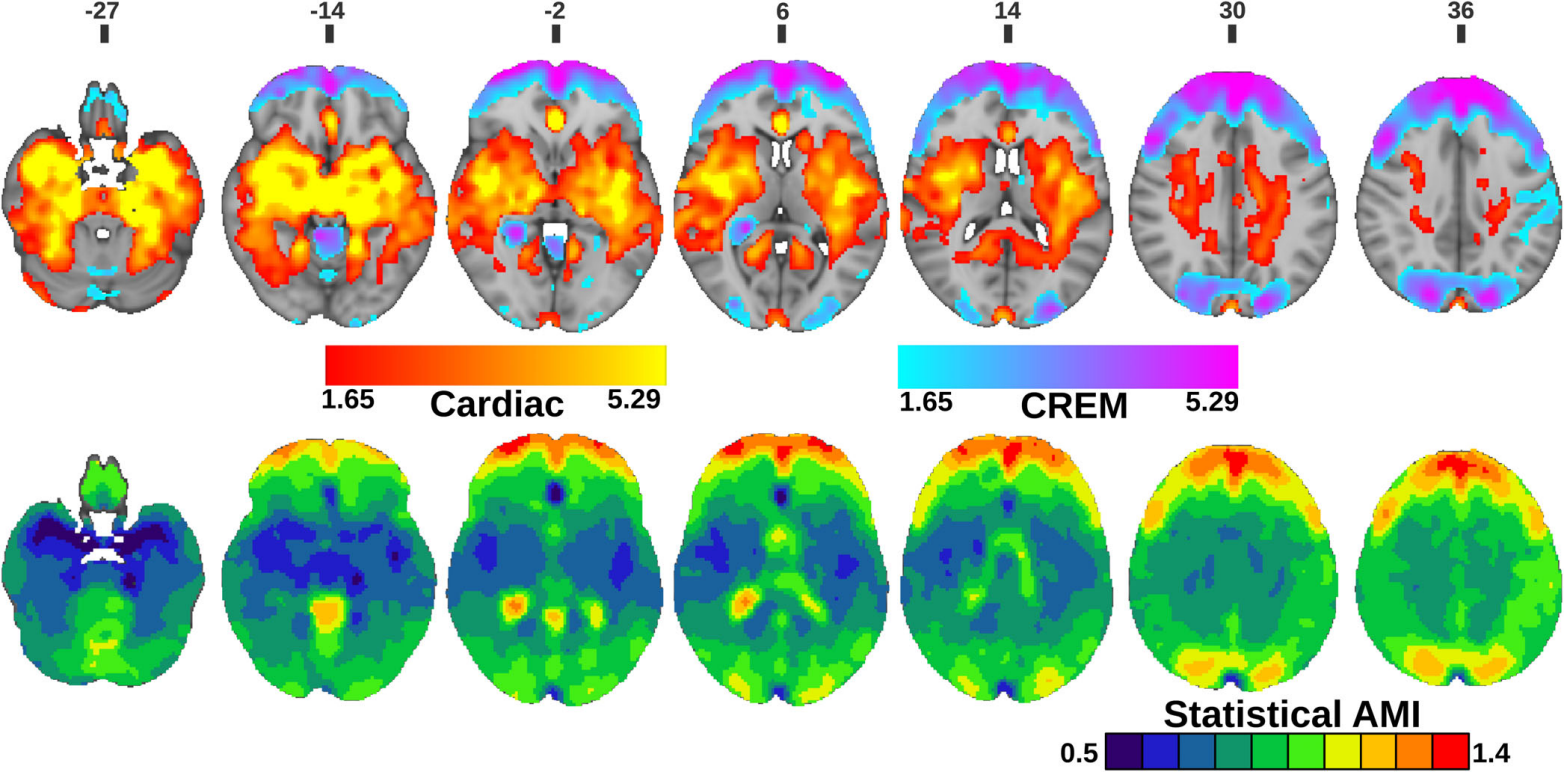
(Top) Statistical comparison between amplitude of cardiac fluctuation (AF_card_) and amplitude of cardiorespiratory envelope modulation (AF_CREM_). Colors in the maps represent where AF_card_ (Red) or AF_CREM_ (Purple) are significantly higher. (FDR corrected, *p* < 0.05 with respective z‐score encoding) (Bottom) Amplitude modulation index (AMI) images. Binary image of statistically significant brain regions with threshold between 0.5 and1.4 with 0.1 increments (*p* < 0.05, FDR corrected)

Voxel‐wise comparisons between different AF maps were performed by a two‐sample *t*‐test using a paired nonparametric threshold‐free permutation test (5,000 permutations) implemented in *vlisa_twosample* from LIPSIA (Lohmann et al., 2018). We used a whole brain mask including white matter, gray matter and CSF. The voxel‐based statistical tests were corrected for the family wise error rate at a significance level of *p* < .05. First, we compared vasomotor AF_VLF_ maps to the other maps (Figure 5) and then compared the respiratory AF_resp_ with the cardiac driven AF_card_ and AF_CREM_ (Figure 6). Finally, we compared within cardiovascular pulsations the AF_card_ and AF_CREM_ maps (Figure 7).

For AMI mapping, we calculated average AMI maps to determine the lowest possible AMI value (0.5), which was then used as a threshold for individual subjects' AMI maps. Statistical testing was done over the threshold value and then increased in increments of 0.1 until the test did not return any significant voxels. A set of binary AMI maps was created to show statistically significant brain regions for each threshold (Figure 7). For statistical testing, the one‐sample t‐test was performed using non‐parametric threshold‐free permutation tests (5,000 permutations) implemented in *vlisa_onesample* from LIPSIA (Lohmann et al., 2018).

**FIGURE 8:**
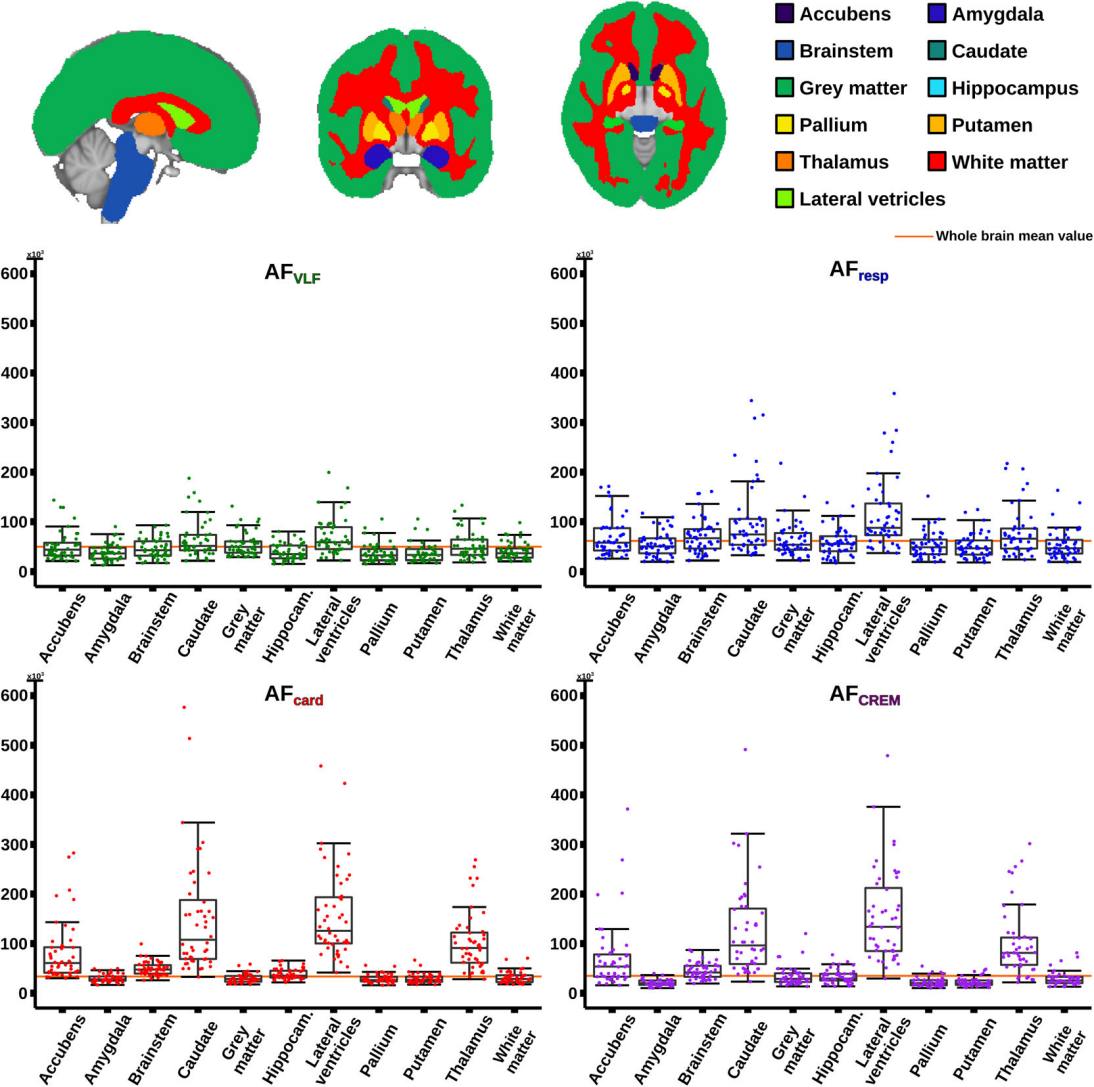
Quantitative analysis of 11 Oxford‐Harvard subcortical atlas regions of interest (ROI) from amplitude fluctuations (AF) (very low frequency (VLF), respiratory, cardiac and cardiorespiratory envelope modulation (CREM), with same color coding as in Figure 3). Data indicating low variability from each of the 11 ROIs (top) for each AFs are presented as box plots (bottom). Orange line indicates the whole brain mean AF of the boxplot

To understand interindividual variability between subjects, the AFs and AMI maps were segmented into 11 anatomical regions using the Harvard‐Oxford subcortical structural atlas as implemented in the FSL toolbox, with threshold of 0.5. These regions were used as ROIs in which average voxelwise AF and AMI values were calculated for every subject. Results were plotted using *geom_boxplot* function in R library *ggplot2, (*Figure 8).

### 2.6 QPP analysis

**FIGURE 9:**
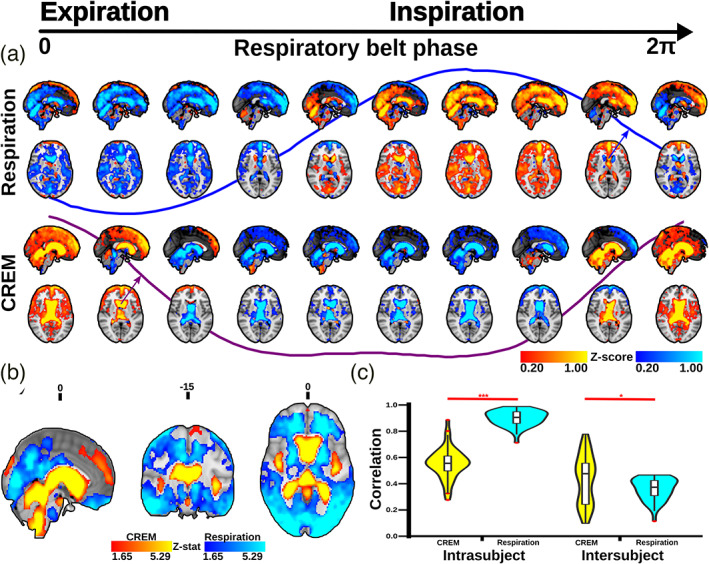
Quasi‐periodic patterns (QPP) waves: (a) 3D time lapsed, group averaged, and phase‐matched QPP waves of respiration and cardiorespiratory envelope modulation (CREM) pulsations triggered to the respiratory belt inspiratory maximum. The color bars indicate group average normalized z‐score values (b) Brain regions significantly different with respect to spatial distribution of amplitude between respiration and CREM QPP waves (*p* < .05, FDR corrected). The color bars indicate the z‐stat values. Red denotes higher amplitude in CREM and blue indicates higher amplitude in respiration (c) Violin plots show the average spatial correlation between intra‐ and inter‐subject level correlations of respiration and CREM. Student *t*‐test: * *p* < .05, *** *p* < .001

MREG data were band‐pass filtered using AFNI *3dTproject* on individual respiratory and cardiac bands, including the upper and lower modulation sidebands. The cardiorespiratory modulation CREM‐signal was derived from the cardiac ± sideband signal for every voxel using MATLAB *envelope* (Figure 1). A modified quasi‐periodic patterns (QPPs) algorithm was used to detect respiratory and CREM brain waves and average them to yield subject‐level averaged QPP maps and to evaluate how they differed in their pulse propagation patterns and amplitude (Kiviniemi et al., 2016; Majeed et al., 2011). The subject‐level maps were timed to start from the beginning of expiration to the end of inspiration, synchronized to the individual subject scanner respiration belt data from each of 38 individuals. A group‐level averaged QPP map was produced by interpolating subject‐level average QPP maps into the same 100 timepoint (10 s) waves for a round number. All subject‐level maps were shifted to the same phase using MATLAB *circshift*. For purposes of display, the group average maps were z‐scored and interpolated to 0.5 mm resolution in MNI space (c.f. Figure 9a).

To test for statistical differences in the amplitudes between subject CREM and respiratory QPP maps, we first calculated individual amplitude maps for each subject by reckoning the maximum difference in the subject's QPP pulsation for every voxel. The resultant amplitude maps were normalized by dividing the amplitude of each voxel by the global mean amplitude of the subject. The CREM and respiration normalized amplitude maps were compared using a paired *t*‐test using the LIPSIA function *vlisa_onesample* (Figure 9b).

To study intrasubject variability, we correlated pulsation waves detected by the QPP algorithm to the subjects' individual average QPP map. Intersubject variability was calculated by correlating subject‐level average maps to the group average map, using the MATLAB *corrcoef* function. Intralevel and interlevel CREM and respiration correlation coefficients were compared statistically using the nonparametric two‐sided, paired‐samples Wilcoxon signed rank test in MATLAB *signrank* (c.f. Figure 9c).

## 3 RESULTS

### 3.1 Physiological pulsation amplitudes: AF_VLF_ , AF_resp,_ and AF_card_

In general, all physiological pulsation amplitudes were strong in CSF spaces, but AF_card_ signals were especially pronounced in CSF spaces and brain areas close to major cerebral arteries and venous sinuses, while AF_VLF_ and AF_resp_ signals were more widely and homogeneously spread in the brain tissue (Figure 3). The AF_VLF_ was dominant in frontal and posterior midline cortical structures, whereas the respiratory AF_resp_ dominated in regions such as cerebellum, basal frontotemporal cortex, white matter, and thalamic structures.

In the WTA‐analysis, AF_VLF_ dominated in cortical gray matter areas. Respiration waves had the widest spatial distribution, being absent only from the ventricles, thalamus, areas and arterial lumen (Figure 4). The AF_resp_ signal was significantly (*p* < .05, FDR corrected) larger than AF_VLF_ and AF_card_ amplitudes in the entire white matter, basal gangliae, bilateral hippocampus and amygdala, brain stem, and cerebellum (Figures 4, 5, 6, 7).

The AF_card_ was significantly larger than both AF_VLF_ and AF_resp_ in central CSF spaces and near major cerebral arteries (Figure 4). The AF_VLF_ exceeded AF_card_ but not AF_resp_ across the neocortex, that is, respiration and VLF power were of very similar amplitude in cortical gray matter. In WTA analysis, AF_VLF_ tended to dominate over otherwise prominent respiration signals in posterior default mode areas and in the right frontal cortex (Figures 4, 5, 6, 7).

### 3.2 Cardiorespiratory envelope modulation—AF_CREM_

The spatial distribution of the respiratory‐driven CREM modulation naturally matches the modulated cardiac pulsation distribution in frontal, lateral, and midline ventricular CSF spaces and perivascular structures along the sinus rectus and major cerebral arteries, (Figure 3). Additionally, the quadrigeminal cisterns had distinct CREM pulsations. Notably, in the statistical and WTA analyses, the AF_CREM_ amplitude was significantly larger than any other physiological pulsation source in the most central CSF spaces of the lateral and third ventricles, and the cerebral aqueduct (Figures 4, 5, 6, 7). In the posterior fossa, the CREM dominated in the quadrigeminal cisterns and at the bottom of the fourth ventricle (Figures 5 and 8). The cardiac pulsation dominated in CSF at the outer edges of the lateral ventricles and upper parts of the fourth ventricle.

When normalized to the cardiac amplitude, the AMI was lowest (0.5, i.e., 50% of the cardiac pulse amplitude) in the areas with strong cardiac pulses and increased proceeding towards the cortex, especially in frontal and occipital parts of both the cerebrum and cerebellum (Figure 7). Also, posterior parts of the lateral ventricles at trigonum regions and the midline near the pineal gland, the AMI map shows high intensity (c.f. Figure 8). It is noteworthy that the degree of modulation of the cardiac pulsations exceeded 50% everywhere in the brain, and even exceeded 100% in frontal, occipital, and trigonal ventricular areas, that is, red areas in Figure 7.

We performed ROI‐based analyses to quantify interindividual pulsation amplitude variances; the intersubject variation proved to depend on anatomical location and frequency, c.f. Figure 8. While there were some outliers, the variance was insufficient to explain the amplitude differences between frequency bands or anatomic areas. AF_VLF_ showed the smallest variation and did not seem to have any distinct areas with a higher level of inter‐individual variation. AF_resp_ has moderate variation in general, while AF_card_ and AF_CREM_ showed a higher range of intersubject variation in the caudate nucleus, thalamus, and lateral ventricles. The mean amplitude of pulsation exceeded the whole brain average in those regions, but were below the global mean in GM, WM, and the bilateral amygdala, putamen and pallidum in all frequency bands (Figure 8).

### 3.3 QPP propagation analysis of CREM and respiratory brain pulsations

QPP analysis showed that on average there was a wave of cardiorespiratory modulation of arterial pulsation amplitude that propagated through the human brain following a unique pattern, distinct from the patterns of vasomotor and cardiorespiratory brain pulsations, both temporally and spatially (Kiviniemi et al., 2016). The CREM wave shares the frequency distribution of the modulating respiration, and we therefore compare these two pulsation sources.

The most obvious difference between the respiratory and CREM pulsations was the phase differences with respect to respiration itself. During inspiration, the respiratory brain BOLD signal intensity increased in cortical areas, and during expiration it declined symmetrically in a nearly sinusoidal manner (c.f. Figure 9a). In the CREM wave, the envelope peak (i.e., cardiac pulsation amplitude maximum) occurred at the crossover from inspiration to expiration (c.f. Figure 9a). Notably, the opposite change (from expiration to inspiration) was associated with a relatively longer nadir in the CREM wave: the cardiovascular pulsation envelope remained low for 60% of the time, while the cardiovascular pulsations peaked only for a relatively short period (40%) over the respiratory cycle (c.f. Figure 9a). For 3D visualization of the differences between CREM and respiratory pulsations, please see also supplementary video 1.

In spatial analysis, the CREM and the respiratory waves differed significantly from each other (Figure 9b). The respiratory pulsation induced a relatively peripheral pulse affecting predominantly the cortical gray matter and entire white matter, while the CREM was more dominant in the areas of strong cardiac pulsatility in, major vessels CSF conduits, and in the ventricles. The spatial similarity/stability of CREM and respiratory waves also differed significantly; within subjects the mean correlation of each CREM wave was 0.540, while with respiratory pulsations the correlation was significantly higher .882 (*p* < .001). The intersubject spatial similarity, on the other hand, had a higher correlation in CREM pulsations (0.413) versus respiratory pulsations (0.342; *p* < .05) (Figure 9c).

## 4 DISCUSSION

Several findings in the present study contribute to a better understanding of physiological brain pulsations. Due to the critical 10 Hz whole brain sampling rate, the current study offers a new view on physiological sources affecting whole brain BOLD signals in the absence of aliasing. The extended frequency spectral analysis revealed a new form of physiological brain contrast, CREM, which illustrates how strongly respiration modulates the amplitude of cardiovascular brain pulsations. We used the ALFF method to map the spatial distribution of all fundamental physiological signal sources in the brain and compared their amplitudes relative to each other.

### 4.1 Spatial distributions of physiological pulsations

Our AF_VLF_ maps showed dominance in peripheral cortical gray matter regions that are virtually identical to findings in previous resting‐state ALFF BOLD studies (Huotari et al., 2019; Yu‐Feng et al., 2007; Zou et al., 2008). The AF_resp_ maps extended over the whole brain, being especially pronounced in the periventricular white matter, cerebellar, and midline structures, as noted previously by Windischberger and co‐workers in 2002 (Windischberger et al., 2002). The AF_card_ maps dominated in major arterial venous, perivascular, and frontal CSF spaces, which has also been detected previously by multiple groups (Dagli, Ingeholm, & Haxby, 1999; Kiviniemi et al., 2016; Tong & Frederick, 2014; Weisskoff, Chesler, Boxerman, & Rosen, 1993).

As far as we are aware, the relative differences of the pulsation amplitudes have not previously been statistically quantified throughout the brain. Our results indicate that the cardiac pulsation dominates in the major cerebral arteries and tissue areas surrounding them, in the CSF ventricles, and the venous sinuses. Interestingly, the CREM had a larger power than its carrier wave, the cardiac pulsation, in most central areas of the CSF ventricles, and in the frontal and occipital cortices (Figures 4, 5, 6, 7). Spatially the most dominant source was the respiratory pulsation, which extended from the periventricular white matter all the way to the cortical gray matter (Figures 4, 5, 6, 7, 8). In cortical gray matter, respiratory and VLF were equally strong sources of the BOLD signal, with no statistically significant difference between them (Figure 5). Interestingly, in a winner takes all map, the VLF dominated in the default mode areas, while in the areas of primary sensorimotor areas resembling task positive areas, it was the respiration that tended to dominate. As previously shown, slow 0.03 Hz variations in respiration (RVT) depth correlate with BOLD signal in the very same gray matter areas (Birn, Diamond, Smith, & Bandettini, 2006; Wise, Ide, Poulin, & Tracey, 2004). The present results suggest that the respiratory brain pulsations can compete as a source of signal variance equally with VLF BOLD signal in the gray matter and are strongly present in all parts of the brain.

### 4.2 Cardiorespiratory amplitude modulation in brain BOLD signal

To the best of our knowledge, this is also the first study to quantify respiratory modulation of cardiovascular brain pulsation amplitude, namely the CREM, in the human brain. The described modulation has been previously detected in fMRI research, but it had not been investigated in depth. The interaction between respiratory and cardiac frequency pulsations were previously demonstrated for spinal canal CSF and jugular venous blood flow in other critically sampled BOLD fMRI studies (Brooks et al., 2008; Friese, Hamhaber, Erb, & Klose, 2004). In the EPI scan, the modulation has been studied with respect to the retrospective image correction (RETROICOR) method, which revealed it to be a significant source of physiological noise in the brain stem (Harvey et al., 2008). However, when modeling physiological noises using interleaved echo‐planar imaging (EPI, TR = 2,800 ms) sequences at the whole‐brain level, the modulation had not been captured accurately, as it was only present in a small number of voxels (Beall, 2010).

Physiologically, the respiratory pulsations modulate the cardiac pulsations of the arterial blood pressure, heart rate, and stroke volume inside the thorax based on the closely intertwined relationship between breathing and circulation (Larsen, Tzeng, Sin, & Galletly, 2010; Lewis, 1908). Based on this modulation, respiration pulses can thus also be quantified from fingertip arterial pulsation data with photoplethysmography (PPG) data, without requiring two separate measures for both cardiorespiratory signals (Charlton et al., 2017; Karlen, Raman, Ansermino, & Dumont, 2013). The PPG results can reflect the microvascular tissue blood oxygenation modulations caused by respiration driven intrathoracic pressure oscillations similar, which are analogous to what BOLD signal can measure from within the brain (Meredith et al., 2012; Nitzan, Faib, & Friedman, 2006). The PPG literature suggests that the CREM signal originates mainly from the intrathoracic flow modulations.

In terms of the intracranial space, the CREM signal seems to be more complex. If the source of CREM modulation were only arising from the thoracic pressure oscillations, it should then be a uniform phenomenon throughout the brain, or at least it should directly follow respiratory induced brain pulsations. However, the present statistical AMI maps show that the cardiac pulsations are least modulated within large arteries and most modulated in the frontal cortex, CSF dorsal horns of the lateral ventricles, and in occipitoparietal areas (Figure 7). The QPP analysis further shows that CREM moves as a wave over the brain, starting from the posterior fossa close to fourth ventricle rather than arising in an arterial area where the modulated cardiovascular pulse first arrives in the brain.

Recent work shows that respiratory inhalation drives venous outflow from brain and also induces a counterbalancing CSF inflow, exactly following the Monro–Kellie doctrine of relative compartment changes inside the incompressible cranium (Dreha‐Kulaczewski et al., 2017; Vinje et al., 2019). And indeed, the CREM wave moves forward from the posterior fossa along the trajectory of CSF inflow into the cranium from the spinal canal (see also supplementary video 1). However, the respiratory pulsation propagates in a completely different manner than the CREM wave (Figure 9). This indicates that the CREM manifests as a unique physiological entity inside the cranial space. While the cardiovascular intra‐arterial pulsations are first modulated inside the thorax directly by the respiration, inside the brain tissue and CSF spaces, the cardiovascular pulsations become further modulated by the inflowing CSF/venous blood pulsations also driven by respiratory changes inside the spinal canal and jugular veins.

### 4.3 The source of physiological BOLD signal fluctuations

The pulsatile and propagating nature of the cardiac and respiratory waves and as well as the modulatory CREM pulsation along brain and CSF structures suggest that they are distinct physiological phenomena (Birn et al., 2006; Huotari et al., 2019; Kiviniemi et al., 2016;Windischberger et al., 2002; Wise et al., 2004). The arterial pressure impulse becomes absorbed into a convective force, pushing blood within the vasculature and CSF along paravascular spaces (Mestre et al., 2018; Rajna et al., 2021). The cardiovascular pulses dominate around arteries, where respiratory modulation is of small but detectable magnitude (Berger, 1901; Mestre et al., 2018; Santisakultarm et al., 2012). The cardiovascular impulses accelerate water protons, causing them to drop momentarily their regional proton spin coherence within both arteries/arterioles and the paravascular (glymphatic) space. The momentary BOLD signal drop can be then detected as a cardiovascular pulse propagating within the brain (Kiviniemi et al., 2016; Posse et al., 2013; Rajna et al., 2019).

During inhalation, deoxygenated venous blood is drained from brain, which increases the BOLD signal (Windischberger et al., 2002; Wise et al., 2004). At the same respiratory phase, CSF flows into the intracranial CSF spaces to compensate for the reduced venous blood volume, as dictated by the Monro–Kellie doctrine stating that any increase in volume of one of the cranial constituents (blood, CSF, or brain tissue) must be compensated by a decrease in volume of the another (Mokri, 2001). The combined effects of the CSF inflow/venous blood outflow together induce the propagating respiratory brain pulsations previously detected using fast MRI scans (Kananen et al., 2018, 2020; Kiviniemi et al., 2016).

Respiration is also a source of rigid body head motion in BOLD scans, especially along the MRI bore B_0_‐field z‐direction, but rigid body motion‐related changes dominate in susceptibility gradient areas at the edges of the brain. However, bulk motion does not follow CSF spaces in the manner of respiratory pulsations, nor does it follow arterial paths, as do the cardiovascular brain pulsations (Figure 3). Therefore, the respiratory brain pulsation seems to be a flow‐dependent effect. Respiration also induces dynamic variations in bulk magnetic susceptibility via thoracic volume changes inside the MRI bore (Glover, Li, & Ress, 2000; Noll & Schneider, 1994). We minimized these susceptibility issues and magnetic field inhomogeneities caused by respiration through the use of dynamic off resonance correction of k‐space (DORK) for MREG data during image reconstruction, and then used AFNI *3dDespike* to remove excess high spikes from the data in addition to applying standard MCFLIRT motion correction (Pfeuffer, de Moortele, Ugurbil, Hu, & Glover, 2002; Zahneisen et al., 2014).

Let us suppose that the source of the respiration signal and CREM were both caused by rigid body head motion. The displacement of a voxel due to respiratory motion would then instantaneously affect the amplitude of both cardiorespiratory pulsations over the whole brain. If rigid body motion were the only source, then both respiratory and the CREM pulsations should have their highest amplitudes in the same voxels, with identical temporal waveforms. This is clearly not the case (Figure 9). However, the pulsations quantified by QPP indicate that the respiratory and CREM pulsations have different spatial distribution and temporal dynamics, as seen in Figure 9a,b. There are also differences in intrasubject and intersubject level pulsation variability (Figure 9c). These results shows explicitly that respiration and CREM waves cannot be both solely induced by a bulk head movement.

### 4.4 The physiological sources of global BOLD signal

The results of this study are in line with previous findings that there are several physiological sources contributing to the global BOLD signal (Liu et al., 2017; Power et al., 2017). The direct respiratory‐induced brain pulsations are themselves a strong source of global signal (see Figures 4, 5, 6, 7 for spatial distribution of respiratory pulsations and supplementary Figure 2 for group average global signal FFT spectra). In a largely overlapping area over the whole gray matter, respiratory power equals the VLF power that is related to neuronally coupled, classical BOLD signal activity (Bandettini et al., 1992; Zou et al., 2008). Although the cardiac pulsations and CREM dominate in CSF and vascular areas, they also affect the signal widely across the brain and affect the global signal.

In order to improve specificity of BOLD signal to neurovascular activation or in functional connectivity studies, the physiological signals should be cautiously removed from the cortical VLF BOLD signal (Birn et al., 2006; Chang, Cunningham, & Glover, 2009; Glover et al., 2000; Wise et al., 2004). However, the physiological signals are repeatedly propagating as waves over the entire brain (see also supplementary video 1), each having a nonuniform amplitude and phase distribution over the entire brain (Chen et al., 2020; Kiviniemi et al., 2016; Yousefi, Shin, Schumacher, & Keilholz, 2018). Thus, the physiological pulsations cannot be removed simply by regressing; any single global signal regressor have same instantaneous phase in virtually each voxel, which is not correct assumption for moving pulsation, and multiple nuisance regressors would excessively reduce the degrees of freedom in the data (Beall, 2010; Chen et al., 2020; Glover et al., 2000). We agree with both Glover and Beall, that a more feasible way to control for physiological signals over the entire brain is presented by applying voxel‐wise corrections with Fourier‐based filtering (Beall, 2010; Glover et al., 2000).

In order to completely remove physiological signals, or to analyze them comprehensively, it is necessary to sample BOLD signal at sufficient frequency, with TR < < 500 ms (Chen, Polimeni, Bollmann, & Glover, 2019). The principal cardiovascular and respiratory pulsations, their harmonics, and furthermore the interactive modulations of the pulsations such as CREM, as shown in this study, are irretrievably aliased in the BOLD signal if the data is sampled too slowly (Huotari et al., 2019). However, recently developed fast fMRI sequences, such as the MREG used in this study, can capture the whole brain volume with partial k‐space sampling at a TR of 100 ms or less. This fast scanning brings a certain penalty in spatial resolution, but on the other hand enables the clear separation of physiological noise sources, removes aliasing, and avoids slice‐timing problems of interleaved scanning (Huotari et al., 2019). This improved signal precision enables stronger statistical inferences and provides a more accurate view of emerging metrics on pathophysiological pulsation mechanisms of human brain (Helakari et al., 2019; Jahanian et al., 2015; (Kananen et al., 2018, 2020; Makedonov et al., 2016; Rajna et al., 2019, 2021; Tuovinen et al., 2020).

## 5 CONCLUSIONS

In summary, the extended spectral analysis of high temporal resolution BOLD data revealed multiple sources of the BOLD signal, each with unique and characteristic dynamic distribution over the brain. In the neocortex, the respiratory pulsations can dominate over the classical low frequency BOLD signals that are attributed to neuronal activity. Our results suggest that respiratory brain pulsations can compete as source of signal variance equally with VLF BOLD signal. Thus, careful separation of cardiorespiratory signal using fast fMRI is strongly recommended for future neurovascular as well as physiological brain signal studies. In addition, the strong respiration pulse introduces a uniquely propagating wave of modulation of the purely cardiac brain pulsations in the brain. Our study identifies multiple sources of global BOLD signal and supports the use of fast fMRI data for their comprehensive separation.

## CONFLICT OF INTEREST

The authors declared no potential conflicts of interest with respect to the research, authorship, and publication of this article.

## Supporting information

**Supplementary figure 1** Group average amplitude modulation index (AMI) maps

**Supplementary figure 2** Group average amplitude spectrum of global BOLD signal from 0.008 to 5.0 Hz. Please note that the at group average FFT spectra the power peaks are widened due individual variability in cardiorespiratory rates in comparison to individual global image power spectrum shown in Figure 1

**Video 1 Quasi‐periodic patterns (QPP) video:** 3D time lapsed group averaged and phase‐matched QPP maps of respiratory pulsations and cardiorespiratory envelope modulation (CREM) triggered to respiratory belt inspiratory maximum. The color bars indicate group average normalized z‐score value. Top video is respiratory, and bottom is CREM pulsation

## Data Availability

The data that support the findings of this study are available on request from the corresponding author. The data are not publicly available due to privacy or ethical restrictions.
