# Supplementary figures and images for: Spectral analysis of physiological brain pulsations affecting the BOLD signal
Source: Hum Brain Mapp. 2021 May 26;42(13):4298–313. doi: 10.1002/hbm.25547 (PMC8356994; doi:10.1002/hbm.25547)

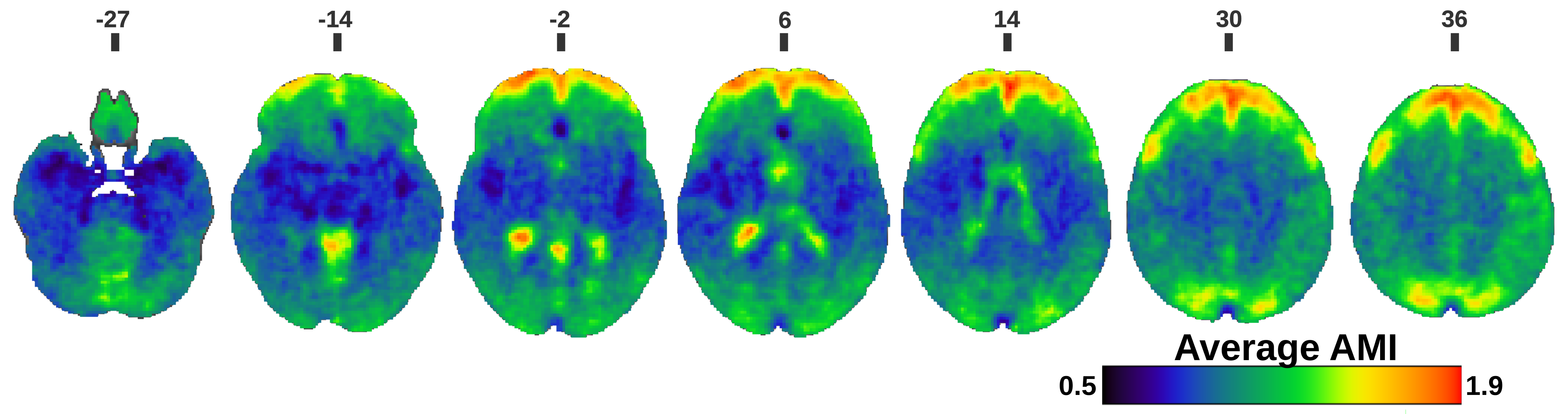

Supplement: Supplementary file 1 — Supplementary figure 1 Group average amplitude modulation index (AMI) maps [file HBM-42-4298-s003.png]

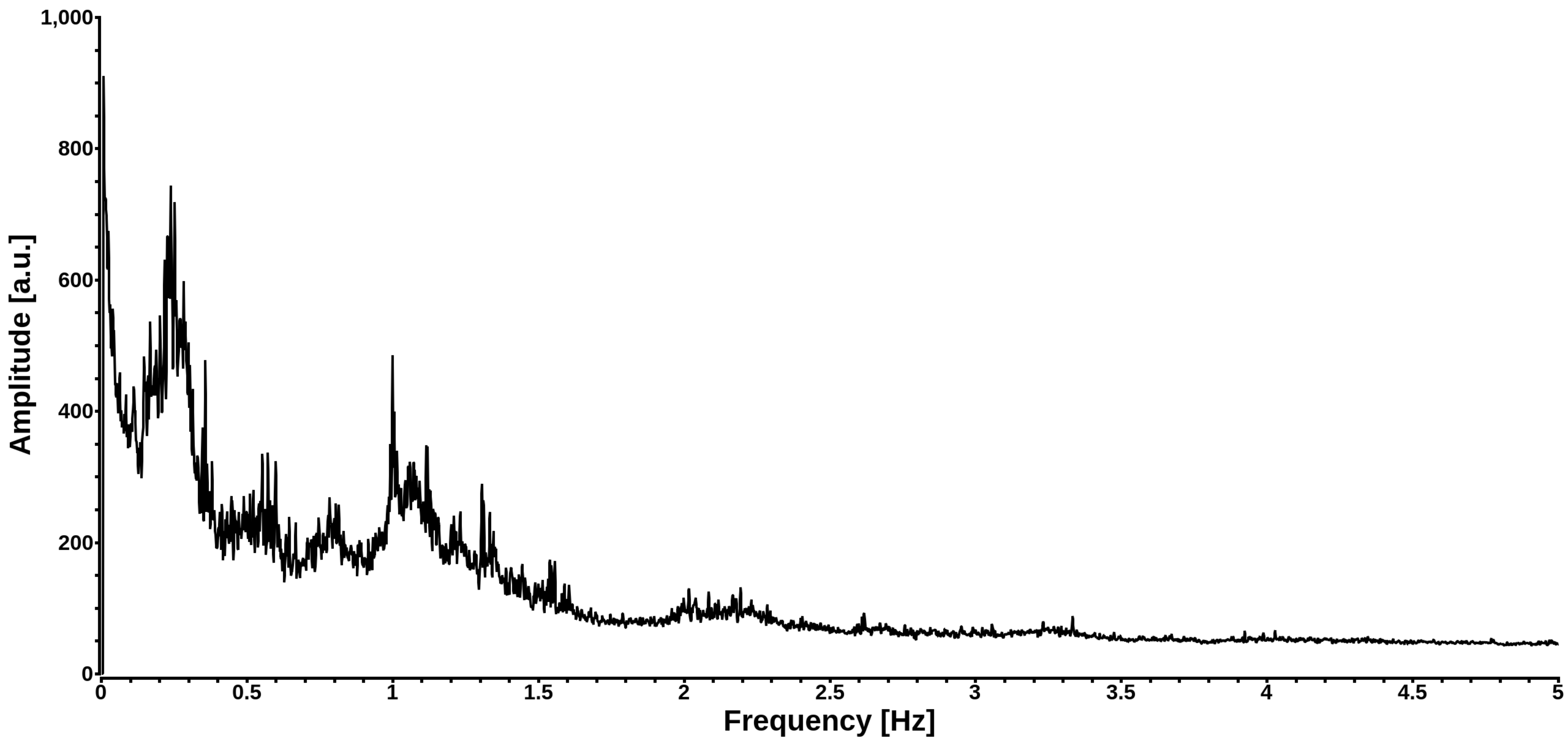

Supplement: Supplementary file 2 — Supplementary figure 2 Group average amplitude spectrum of global BOLD signal from 0.008 to 5.0 Hz. Please note that the at group average FFT spectra the power peaks are widened due individual variability in cardiorespiratory rates in comparison to individual global image power spectrum shown in Figure 1 [file HBM-42-4298-s001.png]
